# The impact of antihypertensive treatment initiation on health-related quality of life and cardiovascular risk factor levels: a prospective, interventional study

**DOI:** 10.1186/s12872-021-02252-7

**Published:** 2021-09-16

**Authors:** Aapo Tahkola, Päivi Korhonen, Hannu Kautiainen, Teemu Niiranen, Pekka Mäntyselkä

**Affiliations:** 1 University of Eastern Finland, Kuopio, Finland; 2 University of Turku, Turku, Finland; 3 Medcare Oy, Äänekoski, Finland; 4 Primary Health Care Unit, Kuopio University Hospital and University of Eastern Finland, University of Eastern Finland, Kuopio, Finland

**Keywords:** Quality of life, Hypertension, Blood pressure, Treatment, Initiation, Cardiovascular, Risk

## Abstract

**Background:**

Effective prevention and treatment of hypertension is one of the most potential interventions in terms of preventing cardiovascular deaths and disabilities. However, the treatment control is often poor. This may be partly explained by the impact of hypertension diagnoses and treatment on health-related quality of life. Quality of life is also an important outcome for a hypertensive patient. Most of the previous studies on health-related quality of life in hypertension have concentrated on patients with treated hypertension and less is known about the initiation of medication and the first treatment year.

**Methods:**

In this interventional study, we followed 111 primary care patients with newly diagnosed hypertension in real world primary care setting in Finland for 12 months.

**Results:**

We found significant decrease in both systolic and diastolic blood pressure levels, as well as modest decrease in cholesterol levels and alcohol consumption. However, the health-related quality of life also slightly deteriorated during the first treatment year.

**Conclusions:**

Our study shows that the initiation of hypertension treatment results in cardiovascular risk decrease among newly diagnosed Finnish hypertensive patients, but it is accompanied by small negative impact on health-related quality of life. However, the deterioration in health-related quality of life is of small magnitude and earlier research demonstrates several measures to enhance treatment and avoid impairment in health-related quality of life.

*Trial registration* ClinicalTrials NCT02377960 (Date of registration: 04/03/2015).

## Background

Poorly controlled hypertension causes numerous preventable deaths and disabilities [1–3]. Inadequate medication adherence and clinical inertia, defined as failure to appropriately initiate or intensify treatment in patients with uncontrolled hypertension, are commonly considered to be among the leading causes for poor hypertension control and represent a major challenge for the medical community [4–8].

The diagnosis and therapy of hypertension are associated with impaired health-related quality of life (HRQoL) which may in turn lead to poor medication adherence [9, 10]. It is important to notice that the negative impact of hypertension on HRQoL may not be inevitable and that in some studies HRQoL of hypertensive patients even improved over time [11, 12]. The underlying causes of these contradictory findings, however, remain unclear. Prior research has indicated that differences in hypertension treatment may affect patients’ HRQoL [11, 13–15]. Another possible explanation is differences in study design. The majority of earlier studies in this field have been cross-sectional with hypertensive patients. However, changes in HRQoL can only be examined in longitudinal studies and are most likely to be detected in patients with newly diagnosed hypertension. A third factor that may explain the prior contradictory findings is the differences between the HRQoL measures. HRQoL has been most commonly measured using the profile-based Short-Form Health Survey (SF-36) [16]. However, studies that have used the preference-based EQ-5D measure have reported EQ-5D scores of hypertensive patients to be quite close to those observed in the general population [17, 18]. Additional evidence from longitudinal studies performed in a real-life setting are therefore needed to better understand the factors affecting HRQoL of hypertensive patients. In addition, limited information of HRQoL changes in newly diagnosed hypertensive primary care patients exists, especially when measured using the EQ-5D.

The aim of this study was to investigate changes in EQ-5D-measured HRQoL and major cardiovascular (CV) risk factors in newly diagnosed hypertensive primary care patients during the first treatment year. We also aimed to examine what factors are associated with the HRQoL changes.

## Methods

This observational study (ClinicalTrials.gov reference NCT02377960) was carried out in primary care setting in Finland [19]. The study was conducted in accordance with the principles of the Declaration of Helsinki and the ethical standards of the institutional review board of the Hospital District of Northern Savo (reference 63/2014). Written informed consent was obtained from all the study patients and study reporting is in line with Consolidated Standards of Reporting Trial (CONSORT) 2010 guidelines.

### Setting

The study was carried out between January 2015 and March 2018. Eight primary care centers in Central Finland were recruited to take part in the study. At the beginning of the study, all centers received basic information about the study and a short lesson on current hypertension treatment guidelines. The study centers included five public sector health centers, one private occupational care center and one public sector health center with occupational health care service.

### Patients

Inclusion criteria for the study patients were: (1) aged between 30 and 75 years, (2) about to start antihypertensive medication for the first time, (3) a clinical diagnosis of hypertension, (4) mobile phone, (4) ability to read Short Messaging Service (SMS) messages, (5) ability to take care of the personal medication, (6) ability to perform home blood pressure (BP) measurements adequately and (7) agreed to use electric drug prescriptions (standard care in Finland). Exclusion criteria were: (1) unwillingness to give informed consent and take part in the study, (2) pregnancy, (3) a malignant disease that was determined to have an impact on life expectancy, (4) having or suspected of having depression or psychosis, (5) atrial flutter or atrial fibrillation, (6) systolic blood pressure (SBP) > 200 mmHg, (7) diastolic blood pressure (DBP) > 120 mmHg, (8) rapid onset or worsening of hypertension, (9) hypokalemia (K < 3.3 mmol/l) or (10) kidney disease, defined as an estimated glomerular filtration rate < 45 ml/min/1.73m2, or proteinuria (albumin-creatinine ratio > 30 mg/mmol, 24-h protein excretion > 500 mg/day, night urine albumin > 200 μg/min, or proteinuria in urine dipstick test). A validated screening tool for depression was part of the study protocol [20]. The flow of the study can be found from our previous article [21]. All patients of the participating study centers who met the inclusion criteria were asked to take part in the study. Study patients were recruited during routine medical appointments by treating physicians when initiating a new antihypertensive medication.

### Baseline measurements

#### Clinical measurements

Patients’ height, weight, office BP and waist circumference were measured by treating physician. Office BP was measured with a Microlife WatchBP Home A or N automatic oscillometric monitor from the left arm after sitting still for at least five minutes [22]. Office BP was defined as the mean of three office measurements. Home BP was defined as the mean of all home measures over a seven-day period (three measurements twice daily, between 6–9 a.m. and 6–9 p.m.). Body Mass Index (BMI) was calculated by dividing the patient’s weight (kg) by the square of his/her height (m). Waist circumference was measured at the midpoint between the lower border of the rib cage and the iliac crest. An electrocardiogram was taken and the following lab tests were performed: fasting plasma cholesterol, fasting plasma glucose, plasma potassium, plasma creatinine and estimated glomerulus filtration rate (eGFR, the Chronic Kidney Disease Epidemiology Collaboration CKD-EPI equation) [23]. Proteinuria was measured with the albumin excretion rate measured with one of the three alternative methods: nightly urine, diurnal urinary protein excretion, or spot urine albumin-creatinine ratio. More detailed information about the clinical measurements can be found from our previous article [21].

### Questionnaires

Basic demographics and other baseline measures were collected immediately after the initial appointment. College- or university-level education was considered higher education. EuroQol five-dimension (EQ-5D, 3L) questionnaire was used to measure HRQoL [24]. EQ-5D covers five dimensions of health: mobility, self-care, usual activities, pain/discomfort and anxiety/depression. Patients gave each dimension a score of one to three, according to three levels of condition severity: “no problems”, “moderate problems” or “severe problems”. The information was then converted into a single EQ-index by applying scores from the British valuation set, with the scale from − 0.59 to 1.00 and the value 1 indicating the best possible HRQoL [24, 25]. In addition, EQ-5D questionnaire includes a standard vertical 20-cm visual analogue scale (EQ-5D VAS) for recording an individuals’ rating for their current overall HRQoL state, with score 0 indicating “the worst imaginable” and score 100 “the best imaginable” health state. Smoking habits were categorized as smoker or non-smoker, based on daily smoking habits. Alcohol use was measured with alcohol consumption questions (AUDIT-C), with a possible score of 0 to 12 [26]. Exercise habits were measured with the Frequency-Intensity-Time (FIT) Index, which has one question each on the frequency, efficiency and duration of exercise [27]. The score range is 1–100, with < 36 indicating low, 37–63 moderate and 64 or more high physical activity. Systematic COronary Risk Evaluation system (SCORE) was used to estimate each study patients’ 10-year risk for a first fatal CV event [28]. The SCORE risk level estimation is based on sex, age, SBP, smoking and total cholesterol level and categorized to low (< 1%), moderate (≥ 1 to < 5%), high (5–9%) or very high risk (≥ 10%). More detailed information about the questionnaires can be found from our previous article [21]

### Interventions and treatment targets

Hypertension treatment in the intervention group was managed by the treating physician and supported by the use of a checklist for initiation of medication and a personalized, unidirectional SMS support for 12 months [19, 21]. The control group received standard treatment for hypertension. Medication decisions in both study arms were made by treating physicians with no study specific medication protocol. The general office BP target was considered to be < 140/90 mmHg for most individuals and < 140/80 mmHg for diabetics, in accordance with the then-current European and Finnish Society of Hypertension guidelines [29, 30]. For home BP, the respective targets were < 135/85 mmHg and < 135/75 mmHg. The general treatment targets for dyslipidemia management were based on 2016 European Society of Cardiology (ESC) and the European Atherosclerosis Society (EAS) Guidelines for the Management of Dyslipidemias [31]. The LDL-C target for treatment for individuals at low or moderate total CVD risk was < 3.0 mmol/l. For individuals at high risk or very high risk, the LDL-C targets were < 2.6 mmol/l and < 1.8 mmol/l, respectively, or a reduction of at least 50% from the base line. However, the treating physicians were able to set different personal BP or LDL targets, if considered necessary. Treating physicians made individual decisions on statin treatment and application of lifestyle consultation for each study patient was carried out without a study-specific treatment protocol.

### Outcomes and data collection

Prior to the final follow-up appointment at 12 months, the study questionnaires were sent by mail to the study patients. At the appointment, all the baseline measures were repeated and the study questionnaires were collected and saved for analyses together with home BP measurements. The medication data from electronic medical records was also investigated and saved for analyses.

### Statistical analyses

The descriptive statistics are presented as means with standard deviation (SD) or as counts (n) with percentages (%). The mean changes (within-individual differences) between the baseline and 12-month measures were assessed using paired t-test or bootstrap type t-test and 95% confidence intervals (CI). In the case of violation of the assumptions (e.g., non-normality), a bootstrap-type test was used (10,000 replications). Effect size (“d”) was calculated by using the method of Cohen for paired samples (mean baseline scores minus mean follow-up scores, divided by the pooled standard deviation) [32]. Effect size of 0.20 was considered small, 0.50 medium, and 0.80 large. Adjusted correlation (partial) coefficients between changes in the EQ-5D and the changes of the characteristics were calculated by the Pearson method, using Sidak adjusted probabilities. Normal distributions were evaluated graphically and with the Shapiro–Wilk W test. All analyses were performed with Stata 16.1 (StataCorp LP; College Station, TX, USA).

## Results

### Baseline characteristics

At 12 months, 94% of the patients (n = 111) had remained in the study (n = 57 in the intervention group, n = 54 in the control group) and were included in the analysis. Baseline characteristics of the 111 study patients are presented in Table 1.

**Table 1:** Baseline characteristics of the 111 study patients

Characteristics	Measures
Female, n (%)	70 (63)
Age, years, mean (SD)	59 (10)
Higher education, n (%)	28 (25)
Married or co-habiting, n (%)	86 (77)
Working, n (%)	53 (48)
BMI, kg/m2, mean (SD)	29.3 (4.8)
Physical activity (FIT-index), mean (SD)	37 (19)
Diabetes mellitus, n (%)	9 (8)
Insulin treatment, n (%)	2 (2)
Lipid lowering medication, n (%)	22 (20)
Any continuous medication other than diabetes, n(%)	47 (42)
Smoking, n (%)	20 (18)
eGFR < 60 ml/min/1,73m2, n (%)	5 (5)
Proteinuria, n (%)	1 (1)
*10-year SCORE risk, n (%)*	
Low	21 (20)
Moderate	66 (62)
High	17 (16)
Very high	2 (2)

BMI, body mass index; eGFR; estimated glomerulus filtration rate; FIT index, Frequency-Intensity-Time (FIT) Index; SCORE, Systematic COronary Risk Evaluation system

### Outcomes

**Fig. 1:**
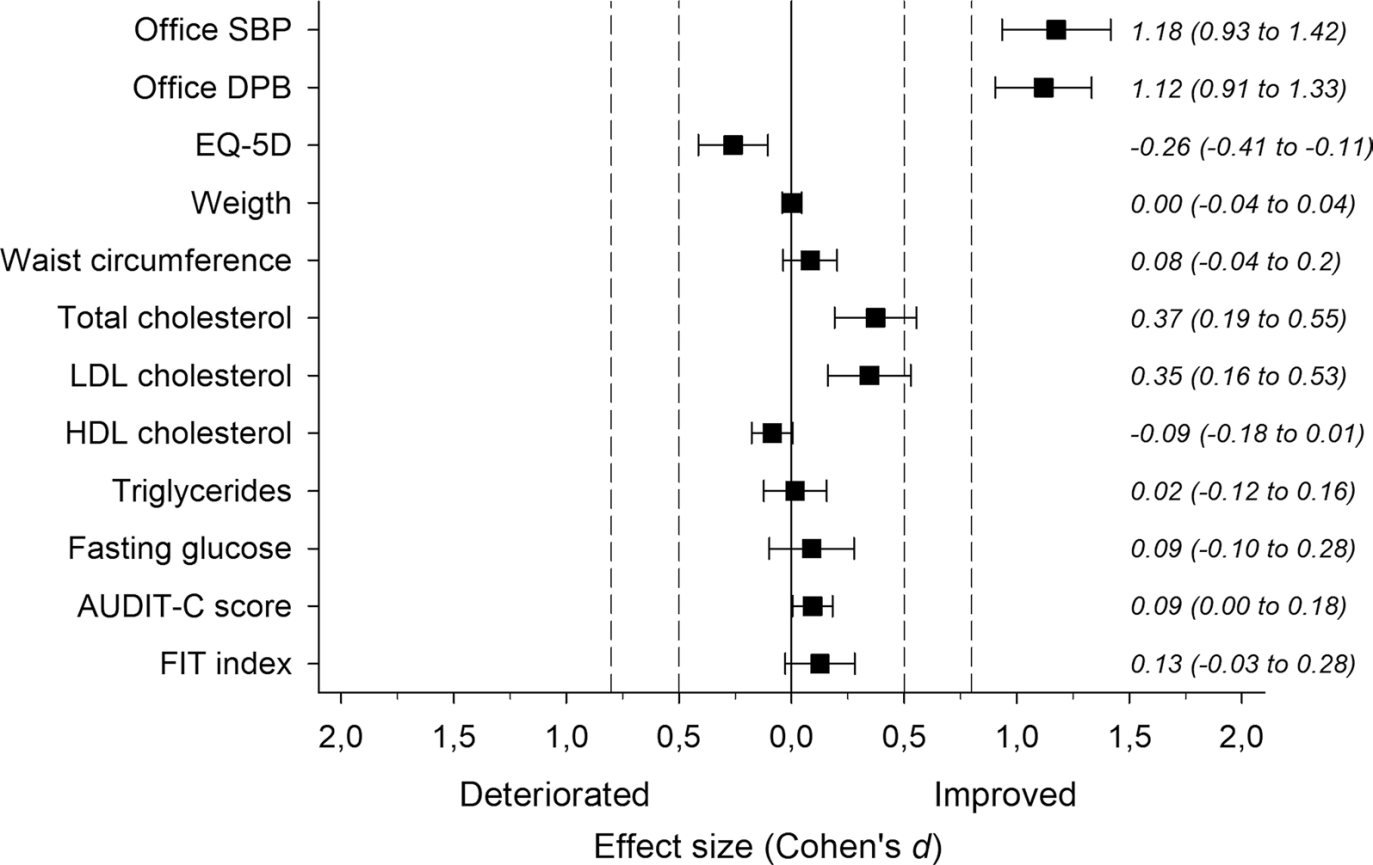
Changes in study outcomes and effect sizes. Effect sizes (Cohen’s *d*) for study outcomes magnitude of change. Effect size of 0.20 was considered small, 0.50 medium and 0.80 large. AUDIT-C, alcohol consumption questions from the alcohol use disorders identification test (AUDIT); DBP, diastolic blood pressure, EQ-5D, EuroQoL questionnaire of health-related quality of life; FIT index, Frequency-Intensity-Time (FIT) Index; HDL, high-density lipoprotein; LDL, low-density lipoprotein; SBP, systolic blood pressure

**Fig. 2:**
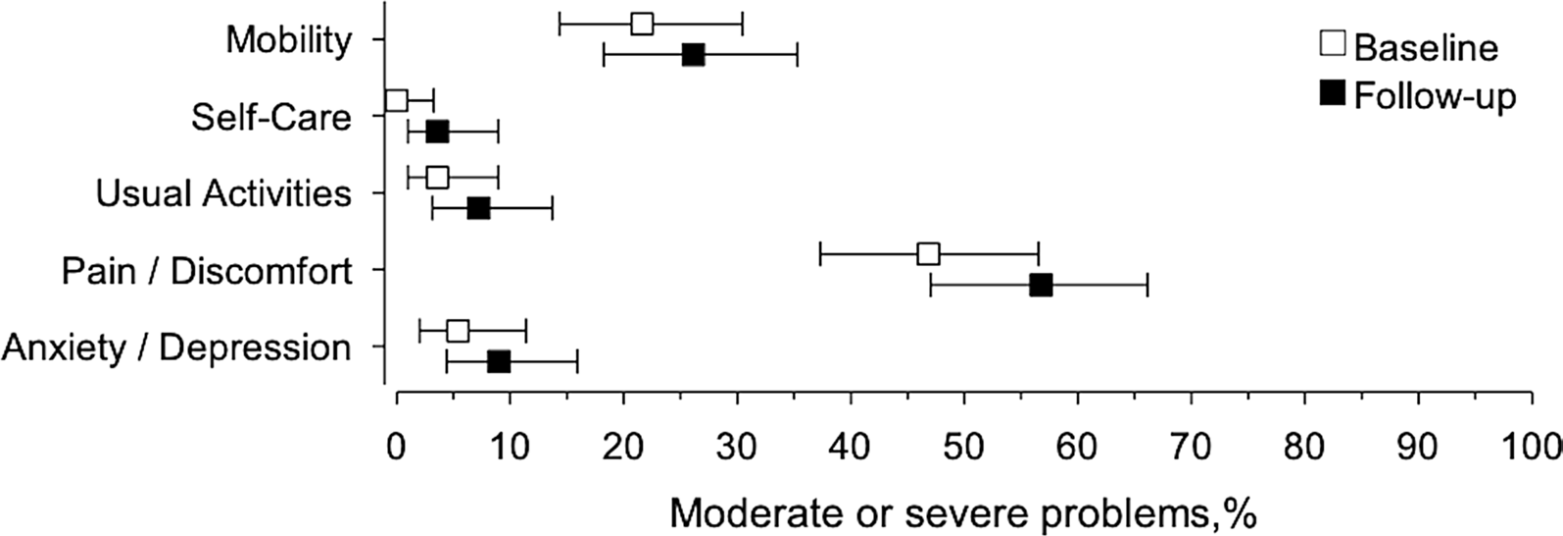
EQ-5D dimensions at baseline and at 12 months. EQ-5D, EuroQoL questionnaire of health-related quality of life

At 12-month follow-up, we found a statistically significant decrease in office SBP, office DBP, home SBP, home DBP, alcohol consumption, total cholesterol level and LDL cholesterol level. Of these changes, only BP reduction was clearly clinically meaningful. During follow-up, four patients quit smoking and one patient started smoking. Any medication change due to a side effect of the antihypertensive medications was made for 25 (23%) patients. At 12 months, 30% of study patients were in systolic office BP target and 36% in systolic home BP target [21]. The EQ-5D index deteriorated and the trend was similar in all EQ-5D dimensions with no significant changes in any single dimensions. However, EQ-5D VAS remained at the same level. Other study outcomes did not change significantly. The changes in study outcomes and effect sizes are presented in Figs. 1, 2 and Table 2. We found no significant correlations between the changes in study outcomes and changes in EQ-5D index. The correlations are presented in Table 3.

**Table 2:** The changes in study outcomes for all participants at 12 months (N = 111)

Outcome	At baseline mean (SD)	Change at 12 months mean (95% CI)	*P*-value
Office SBP, mmHg	173 (20)	− 22 (− 27 to − 18)	< 0.001
Office DBP, mmHg	102 (12)	− 13 (− 15 to − 10)	< 0.001
Home SBP, mmHg	153 (13)	− 15 (− 18 to − 11)	< 0.001
Home DBP, mmHg	92 (7)	− 10 (− 12 to − 8)	< 0.001
EQ-5D	0.872 (0.153)	− 0.045 (− 0.076 to − 0.015)	0.004
EQ-5D VAS	77 (14)	1 (− 1 to 3)	0.32
Weight, kg	84.1 (17.2)	0.0 (0.7 to 0.7)	0.94
Waist circumference, cm	101 (16)	− 1 (− 3 to 1)	0.21
Total cholesterol, mmol/l	5.36 (1.11)	− 0.37 (− 0.56 to − 0.19)	< 0.001
LDL cholesterol, mmol/l	3.15 (1.01)	− 0.32 (− 0.51 to − 0.13)	< 0.001
HDL cholesterol, mmol/l	1.61 (0.45)	− 0.04 (− 0.08 to 0.01)	0.069
Triglycerides, mmol/l	1.37 (0.98)	− 0.01 (− 0.13 to 0.10)	0.82
Fasting glucose, mmol/l	5.87(0.85)	− 0.07 (− 0.23 to 0.10)	0.40
Alcohol use, AUDIT-C score	3.3 (2.6)	− 0.2 (− 0.5 to − 0.0)	0.048
Physical activity, FIT index	38 (19)	2 (–1 to 5)	0.10

AUDIT-C, alcohol consumption questions from the alcohol use disorders identification test (AUDIT); DBP, diastolic blood pressure, EQ-5D, EuroQoL questionnaire of health-related quality of life; FIT index, Frequency-Intensity-Time (FIT) Index; HDL, high-density lipoprotein; LDL, low-density lipoprotein; SBP, systolic blood pressure; VAS, visual analogue scale

**Table 3:** Correlations between the changes in study outcomes and changes in EQ-5D index

Outcome	r (95% CI)	
	Crude	Adjusted*
Weight, kg	0.08 (− 0.11 to 0.27)	0.06 (− 0.13 to 0.25)
Waist, cm	− 0.07 (− 0.25 to 0.13)	− 0.13 (− 0.32 to 0.06)
Physical activity, FIT index	0.05 (− 0.14 to 0.23)	0.08 (− 0.11 to 0.26)
AUDIT-C score	0.02 (− 0.17 to 0.21)	− 0.01 (− 0.20 to 0.18)
Total cholesterol, mmol/l	− 0.05 (− 0.24 to 0.15)	0.01 (− 0.18 to 0.21)
LDL cholesterol, mmol/l	− 0.05 (− 0.25 to 0.15)	0.01 (− 0.19 to 0.21)
HDL cholesterol, mmol/l	0.10 (− 0.10 to 0.29)	0.16 (− 0.04 to 0.35)
Triglycerides, mmol/l	− 0.03 (− 0.23 to 0.17)	− 0.07 (− 0.27 to 0.13)
Fasting glucose, mmol/l	− 0.09 (− 0.29 to 0.11)	− 0.12 (− 0.31 to 0.08)
*Office BP, mmHg*		
SBP	− 0.13 (− 0.32 to 0.06)	− 0.02 (− 0.21 to 0.17)
DBP	− 0.13 (− 0.31 to 0.06)	− 0.11 (− 0.29 to 0.08)

*Adjusted for baseline values of EQ-5D, age and sex

AUDIT-C, alcohol consumption questions from the alcohol use disorders identification test (AUDIT); BP, blood pressure; DBP, diastolic blood pressure, EQ-5D, EuroQoL questionnaire of health-related quality of life; FIT index, Frequency-Intensity-Time (FIT) Index; HDL, high-density lipoprotein; LDL, low-density lipoprotein; SBP, systolic blood pressure

## Discussion

This study demonstrated a decrease in several risk CVD risk factors in Finnish primary health care patients undergoing the first year of hypertension treatment, but also a modest deterioration of patients’ HRQoL. Of all study outcomes, the largest effect size was seen with BP change while other changes in outcomes had effect sizes from trivial to medium. We found no correlation between the degree of change in BP levels and EQ-5D index. Based on our findings, the current standard way to start hypertension treatment in Finland may have negative effect on hypertensive patients’ quality of life, even when the treatment initiation is associated with clinically significant decrease in individuals’ CVD risk.

### Comparison with existing literature

The association between awareness of hypertension and absenteeism was reported as early as 1970s [33]. After that, several studies and systematic reviews have reported correlation between diagnosed hypertension and impaired HRQoL in Finland and internationally [9, 10, 34]. Hypertensive patients seem, interestingly enough, to report low scores especially for the physical component of the HRQoL, which we could not confirm in our study [9, 10]. In accordance to our findings, earlier studies have indicated that the lower HRQoL is associated mainly with the awareness of being sick, i.e. “labeling” and not with the elevated BP or even the side effects of the treatment, per se [34, 35]. Other factors, such as comorbid diseases and the intensity of antihypertensive treatment are also thought to be related to lower HRQoL [36]. In a longitudinal setting, however, the labeling effect seems to fade away and more classical risk factors such as macroalbuminuria, comorbid conditions, smoking, depression and dyslipidemia tend to best predict lower HRQoL [36].

In accordance to earlier studies using EQ-5D instrument, the magnitude of change in HRQoL in our study was quite modest [17, 18]. In our study, the mean VAS scale score (77) and mean EQ-5D index score (0.87) were quite high compared to earlier research findings and only slightly lower than average scores in Finnish and other general populations [17, 37, 38]. A meta-analysis by Dyer and others reported EQ-5D index scores for cardiovascular disease from 0.78 for mild states to 0.51 for moderate/severe states [39]. In 2010, Tsiplova and others reported mean EQ-5D index scores of 0.78 for hypertension [17]. In a recent review by Zhou and others, EQ-5D index scores for hypertension ranged from 0.78 to 0.93 [40].

This leads us into an interesting question of the minimum change in EQ-5D score that is considered relevant. The concept of minimal important difference (MID) can be defined from the patient perspective as ‘The smallest difference in score in the domain of interest which patients perceive as beneficial and which would mandate, in the absence of troublesome side effects and excessive cost, a change in the patient’s management’ [41]. MID in EQ-5D can be estimated by more than one method [17]. Two commonly used methods are “anchor-based” method (MID is calculated based on the minimum score change that is connected to a change in an anchor questionnaire such as self-rated health status) and “distribution-based” method (MID is calculated based on the distribution of the scores in a focus population and a definite cut-point, usually one-half SD, is used) [17, 42]. Often the changes of 0.05 or more are considered meaningful [43]. On this basis, the change in EQ-5D index demonstrated in our study (− 0.045) is on the minimum limit of meaningful change. However, in their foundational study using data from eight longitudinal studies (including individuals with CVD, but not hypertensive patients), Walters and Brazier estimated the mean MID to be 0.074 (range − 0.011 to 0.140). More recently, Tsiplova and others estimated the mean anchor-based MID to be 0.044 and the mean distribution-based MID 0.091 in Canadian population [17].

### Implications for research and practice

The association between hypertension and impaired HRQoL is well established, but it is important to notice that the negative effect of labeling does not seem to be unavoidable. First, the magnitude of deterioration in HRQoL is usually small, as discussed above. Second, the effect of labeling seems not to be permanent and the factors associated with poor HRQoL over time are mostly treatable, classical risk factors such as macroalbuminuria, smoking, depression, and dyslipidemia [36]. Third, HRQoL of hypertensive patients can also improve over time [11, 12]. As for mechanisms behind the change for better, several earlier studies indicate that the adverse effects of labeling can be avoided by more intensive interactions between the patients and their health care providers [11, 14, 44]. Emphasis on lifestyle intervention and especially promoting physical exercise also seem to be elements of hypertension treatment that tend to enhance patients’ HRQoL [11, 13, 15, 45]. Some evidence also indicates that good treatment control, medication adherence and continuity of care are associated with better HRQoL in hypertension [11, 12, 46, 47]. In our study, we could not confirm the positive association between good treatment control and better HRQoL, probably partly due to different assessment method of HRQoL, relatively short follow-up period and insufficient statistical power. It is possible that more intensive interaction with the study patients and more robust lifestyle intervention might have canceled the deterioration of HRQoL in our study, too.

Future studies in this area should prefer longitudinal study designs with a follow-up of at least four years and experimental study designs rather than cross-sectional and observational studies. The aim should be to find interventions and treatment protocols that improve both hypertension control and HRQoL. Especially, we call on experimental studies examining the association of HRQoL and treatment adherence in hypertension.

As for physicians, hypertension should still be considered a major CVD risk factor that should be actively screened and treated. However, it is important to note that hypertension treatment is more than medication and more than just treating the numbers. It is essential to take HRQoL into account and adjust the treatment methods accordingly. Emphasis should be especially put on combining good physicians-patient communication and effective lifestyle intervention combined with determined medication treatment. Poorly treated and poorly controlled hypertension is a double disservice for a patient: The present quality of life compromised for poor protection against the future CVD complications.

## Strengths and limitations

The strengths of this study include the fact that we studied the first treatment year of newly diagnosed hypertensive patients, while previous studies examining the HRQoL in hypertension have usually included patients with treated hypertension. As an interventional, longitudinal study, our study also adds value to more common cross-sectional studies in this area. Furthermore, the study was conducted in a real-life health care environment and the study population was representative of a typical primary care patient population. The study findings are therefore widely generalizable to primary care.

This study also has some limitations. First, the study population was most likely too small to fully detect the changes in the study outcomes. The limited power of the study also precluded the possibility for interesting subgroup analyses concerning, for example, the effect of medication side effects and medication dosage on HRQoL. Second, in our study population, the hypertension treatment did not have impact on the study patients’ waist circumference, weight or physical activity. This may partly explain the perceived deterioration of HRQoL, as earlier studies that have successfully addressed these risk factors, have many times also achieved positive impact on HRQoL [13, 15, 45]. Third, our study lacked a reference group that could have served as the basis for before-after comparisons. Fourth, use of only two time-point measures (baseline and follow-up measures) did not allow us to conduct longitudinal data regression analysis with multiple time points. Furthermore, the base line data with home BP measurement was not complete. Several study patients (n = 35) reported having performed home BP measurements prior to diagnoses of hypertension, but did not bring the measurement data to the clinic. With these patients, the treating physician decided to trust on the patients’ narrative combined with office BP measurements and proceeded to diagnoses and initiation of medication.

## Conclusions

In this study, we demonstrated that treatment of hypertension among newly diagnosed Finnish primary care patients resulted in significant decrease in BP levels and a modest decrease in cholesterol levels and alcohol consumption. However, HRQoL also slightly deteriorated during the first treatment year. Thus, from the patients’ perspective, the onset of hypertension treatment may seem like a trade-off between the future health outcomes and the present quality of life. Health care professionals should actively employ learnings from earlier research to enhance hypertension treatment and avoid HRQoL impairment.

## Data Availability

The datasets generated and analysed during the current study are not publicly available due to protection of individual privacy, but are available from the corresponding author on reasonable request.

## Abbreviations

AUDIT: Alcohol use disorders identification test
AUDIT-C: Alcohol consumption questions from the AUDIT
BP: Blood pressure
CV: Cardiovascular
CVD: Cardiovascular disease
DBP: Diastolic blood pressure
EAS: European Atherosclerosis Society
ESC: European Society of Cardiology
EQ-5D: EuroQoL questionnaire of health-related quality of life
FIT index: Frequency-Intensity-Time (FIT) Index
HDL: High-density lipoprotein
HRQoL: Health-related quality of life
LDL: Low-density lipoprotein
MID: Minimally important difference
SBP: Systolic blood pressure
SCORE: Systematic COronary Risk Evaluation system
SMS: Short messaging service (messages)
VAS: Visual analogue scale
